# Long Non-Coding RNA MDFIC-7 Promotes Chordoma Progression Through Modulating the miR-525-5p/ARF6 Axis

**DOI:** 10.3389/fonc.2021.743718

**Published:** 2021-09-21

**Authors:** Kai Zhang, Zixiang Liu, Zhidong Wang, Zhangzhe Zhou, Xiaofeng Shao, Xi Hua, Haiqing Mao, Huilin Yang, Ke Ren, Kangwu Chen

**Affiliations:** ^1^Department of Orthopedic Surgery, The First Affiliated Hospital of Soochow University, Suzhou, China; ^2^Collaborative Innovation Center of Sichuan for Elderly Care and Health, Chengdu Medical College, Chengdu, China; ^3^School of Laboratory Medicine/Sichuan Provincial Engineering Laboratory for Prevention and Control Technology of Veterinary Drug Residue in Animal-Origin Food, Chengdu Medical College, Chengdu, China

**Keywords:** chordoma, lncRNA MDFIC-7, miR-525-5p, ARF6, proliferation, Warburg effect

## Abstract

**Background:**

Chordoma, an extremely rare malignant tumor, remains difficult to be cured because of its strong local invasiveness and high recurrence rate. Long non-coding RNAs (lncRNAs) have been demonstrated to play multiple roles in various cancers. The purpose of this study was to investigate the modulatory function of lncRNA MDFIC-7 in chordoma and to elucidate its underlying mechanisms.

**Methods:**

Quantitative real-time polymerase chain reaction was performed to detect the expression of lncRNA MDFIC-7 in tumor tissues and adjacent nontumorous tissues collected from 15 chordoma patients, as well as in chordoma cell lines. Gene silencing and overexpression experiments were carried out by RNA interference and lentiviral transduction. The effect of lncRNA MDFIC-7 on the proliferation of chordoma cells was evaluated by cell counting kit-8 assay, colony formation assay and xenograft tumor experiments. RNA immunoprecipitation and dual luciferase reporter assays were conducted to evaluate the binding between lncRNA MDFIC-7 and miRNA-525-5p and the interaction between miR-525-5p and the 3′ untranslated region of ADP-ribosylation factor 6 (ARF6) mRNA. The glycolytic capacity and mitochondrial function of chordoma cells were measured by the Seahorse Bioscience XF96 Extracellular Flux Analyzer.

**Results:**

The expression of lncRNA MDFIC-7 was higher in chordoma tumor tissues than in adjacent non-tumor tissues. Downregulation of lncRNA MDFIC-7 reduced colony formation and cell proliferation in chordoma cells and decreased xenograft tumor growth in a nude mouse model. Moreover, lncRNA MDFIC-7 knockdown attenuated the Warburg effect in chordoma cells and xenograft tumors. LncRNA MDFIC-7 knockdown elevated miR-525-5p levels and decreased ARF6 expressions. Overexpression of ARF6 reversed the inhibitory effect of lncRNA MDFIC-7 knockdown on cell proliferation and the Warburg effect in chordoma cells and xenograft tumors. Mechanistically, lncRNA MDFIC-7, as a molecular sponge of miR-525-5p, negatively regulated miR-525-5p expression and promoted the gene expression of ARF6, a miR-525-5p target.

**Conclusion:**

Our findings demonstrate that lncRNA MDFIC-7 acts as a molecular sponge to competitively bind to miR-525-5p and promote expression of ARF6. The lncRNA MDFIC-7/miR-525-5p/ARF6 axis regulates chordoma progression and the Warburg effect in chordoma, suggesting that lncRNA MDFIC-7 and miR-525-5p could be promising therapeutic targets for the treatment of chordoma.

## Introduction

Chordoma is an extremely rare malignant tumor with an incidence lower than 1 case per 1 million individuals per year. Chordoma originates from embryologic notochord remnants and is most commonly seen in the sacrum and skull base, with some cases in the cervical and thoracolumbar vertebrae (1–3). The primary treatment method for chordoma is surgical resection combined with chemo- or radiation therapy. However, chordoma is difficult to treat by surgery and is often resistant to chemo- and radiotherapy, leading to intensive local invasiveness, recurrence and metastasis (4). The recurrence rate in patients with chordoma is as high as 44% to 78% (5–7), and the 5- and 10-year relative survival rates of chordoma patients in the USA are 67.6% and 39.9%, respectively (8). Therefore, there is an urgent need to understand the molecular mechanism involved in the progression of chordoma to help identify potential novel targets for chordoma therapy.

Long non-coding RNAs (lncRNAs), a type of non-coding RNA with over 200 nucleotides in length, have been demonstrated to play multiple roles in various physiological processes and disease progression, especially in tumorigenesis and metastasis of various cancers (9–11). Although crucial roles of lncRNAs in multiple cancers have been reported in various studies, only a few reports have focused on the function of lncRNAs in chordoma. Xia et al. showed that the lncRNA LOC554202 may play an important role in inhibiting chordoma cell proliferation and invasion through modulating the EZH2/miRNA-31 axis and inactivating the oncogene RNF144B (12). Recently, Guo et al. found that lncRNA LINC00662 may participate in the malignant progression of chordoma by targeting miRNA-16-5p and promoting RNF144B expression (13). Ma et al. reported the potential link between lncRNA KCNQ1OT1 and chemotherapy resistance of chordoma (14), and lncRNA LINC00525 was shown to promote the aggressive phenotype of chordoma through modulating the miRNA-505-3p-HMGB1 axis (7). Zhu et al. demonstrated that lncRNA XIST plays a key role in chordoma progression by regulating the miR124-3p/iAPSS pathway (15), and Zhang et al. predicted the role of several lncRNAs in regulating dural penetration in clival chordoma through analyzing lncRNA and mRNA expression profiles (16). These recent studies confirm the important roles of lncRNAs in the regulation of chordoma progression. However, whether other lncRNAs function in the tumorigenesis of chordoma is unknown. In addition, the underlying molecular mechanisms of lncRNAs in the regulation of chordoma progression remain largely unclear.

In this study, we analyzed the differentially expressed lncRNAs in 15 chordoma patients and found that the expression of lncRNA MDFIC-7 was significantly elevated in chordoma tissues compared to paired adjacent non-tumor samples. The expression of microRNA-525-5p (miRNA-525-5p), a target of lncRNA MDFIC-7 predicted by the online tool of DIANA, was downregulated in tumor tissues. LncRNA MDFIC-7 (NONCODE gene ID: NONHSAG048587.2) is a non-protein coding gene located on human chromosome 7q31.1. No study has determined its role in any cancer. A few reports have been found on miRNA-525-5p. Zhu et al. showed that three miRNAs including miRNA-516a-3p, miRNA-629 and miRNA-525-5p were highly expressed in pediatric systemic lupus erythematosus (pSLE) patients. These three miRNAs might be specific to pSLE and may be used as novel biomarkers of pSLE for diagnose and disease monitoring. However, the detailed function and underlying mechanism were unclear (17). Zhang et al. demonstrated that miR-525-5p plays a role in mediating the invasion of trophoblast cells by regulating HOXD10; miR-525-5p overexpression promoted proliferation, invasion and EMT of HTR-8 cells and regulated pre-eclampsia placenta (18). Moreover, Yang et al. found that androgen receptor can alter the metastasis of prostate and bladder cancer through changing the expression of the vasculogenic mimicry biomarker SLPI by miR-525-5p; therefore, targeting the androgen receptor–miR-525-5p-SLPI axis may be a strategy to suppress prostate cancer metastasis (19). However, the roles of lncRNA MDFIC-7 and miRNA-525-5p in chordoma progression have not been reported.

ADP-ribosylation factor 6 (ARF6) belongs to the ARF protein family of small GTPases, which contains six ARF isoforms (ARF1–6) that are grouped into three classes on the basis of their sequence homology: Class I (ARF1–3), Class II (ARF4–5) and Class III (ARF6) (20). ARF6 is widely expressed in mammalian cells and has a highly conserved sequence. ARF6 is involved in regulating plasma membrane transport and intracellular actin assembly (21). ARF6 has many other roles in regulating multiple physiological and pathological processes, including cell membrane ruffle formation and adhesion, tumor formation, tumor cell proliferation, migration, invasion and metastasis (22–27). Recently, a number of studies have shown that activation of ARF6 and its downstream signaling is essential for the progression of multiple cancers, and overexpression of ARF6 was observed in several cancers and is associated with poor overall survival, including in pancreatic cancer, melanoma, breast cancer, and lung adenocarcinoma (25, 28–33). However, the function of ARF6 in chordoma has not been examined.

In the present study, we sought to clarify the regulatory role of the lncRNA MDFIC-7/miR-525-5p/ARF6 axis in chordoma progression and examine the potential underlying mechanisms. We demonstrate that the lncRNA MDFIC-7/miR-525-5p/ARF6 axis contributes to the tumorigenesis of human chordoma and facilitates the Warburg effect and cancer progression. Our findings will shed light on understanding the mechanisms of chordoma progression and provide potential targets for chordoma therapy.

## Materials and Methods

### Patient Samples

A total of 15 pairs of chordoma tissues and adjacent non-cancerous tissues were collected from 15 chordoma patients in the Department of Orthopedics, the First Affiliated Hospital of Soochow University. All of the enrolled patients had not received chemo- or radiotherapy before surgical excision. The tissues were frozen in liquid nitrogen after resection and stored at -80°C until analyses. All participants signed their informed consents. This study was approved by the ethics committee of the First Affiliated Hospital of Soochow University.

### Cell Culture

The human chordoma cell lines U-CH1 and U-CH2 were obtained from the American Type Culture Collection (Manassas, VA, USA) and cultured in IMDM-RPMI 1640 (4:1) supplemented with 10% FBS (Gibco/Thermo Fisher Scientific, Shanghai, China), 100 U/ml penicillin and 100 mg/ml streptomycin (Biological Industries, HertzliyaPituach, Israel). Cells were cultured in a humidified incubator with 5% CO_2_ at 37°C.

### Plasmid Construction

The full-length coding sequence of lncRNA MDFIC-7 (lnc-MDFIC-7-wt) and its mutant (lnc-MDFIC-7-mut) were inserted into the pmirGLO empty vector to generate pmirGLO-MDFIC-7-WT and pmirGLO-MDIFC-7-mut, respectively. The ARF6 3′-UTR (3′-UTR-wt) and its mutant (3′-UTR-mut) were inserted into the pmirGLO-vector to generate pmirGLO-ARF6-3′UTR-wt and pmirGLO-ARF6-3′UTR-mut, respectively. The pGLO vector, also named pmirGLO, was obtained from Promega (Madison, WI, USA).

For knockdown of MDFIC-7, we used the following shRNA sequence: 5’- GATCCCCGAGAGAGAATTAAAGTCTATTCAAGAGATAGACTTTAATTCTCTCTCTTTTTGGAAA-3’; the control shRNA sequence was 5’- TGCTGAAATGTACTGCGCGTGGAGACGTTTTGGCCACTGACTGACGTCTCCACGCAGTACATTT-3’. The shRNAs were cloned into lentivirus vectors from Addgene (Watertown, MA, USA) to generate Lv-sh-MDFIC-7 and Lv-sh-NC.

The mock, mimic, and inhibitor for miR-525-5p were inserted into lentiviruses. We inserted the full length ARF6 (NCBI Reference Sequence: NM_001663.4) into a lentivirus vector to generate Lv-ARF6, and Lv-vector was used as a negative control.

All lentivirus constructs were synthesized and packaged by GenePharma (Shanghai, China).

### Cell Transfection and Lentivirus Infection

U-CH1 or U-CH2 cells were seeded in dishes or plates and grown to 70% confluence. Plasmids were transfected using Lipofectamine 2000 agent (Invitrogen). The titer of the packaged lentivirus used in experiments was approximately 1.0 × 10^9^ infectious units per milliliter. Cells were incubated with lentivirus for 48 h, and then cells were collected for examination or used in further experiments.

### RNA Isolation, Reverse Transcription and Quantitative Real-Time Polymerase Chain Reaction

Total RNA was extracted from tissues and cells using TRIzol reagent (Thermo Fisher Scientific), and 0.5 μg of total RNA was used for reverse transcription to generate complementary DNA (cDNA) using SMART MMLV Reverse Transcriptase (Takara Bio, Inc., Dalian, China). qRT-PCR was performed with the AceQ qPCR SYBR GreenMaster Mix kit (Vazyme, Nanjing, China) on a CFX96 Real-Time PCR Detection System (Bio-Rad Laboratories, Inc., Hercules, CA, USA). GAPDH mRNA was used as an internal control for quantification of lncRNAs and mRNAs, and the small nucleolar RNA (snRNA) of U6 was used to normalize the relative abundance of miRNA-525-5p. The 2-ΔΔCt method was used to determine gene expression. All experiments were performed in triplicate. The primers used in experiments are provided in the **Supplementary Table S1**.

### Western Blot

Tissues and cells were lysed using RIPA lysis buffer (Thermo Fisher Scientific). Equal amounts of protein (30 μg) were separated by sodium dodecyl sulfate–polyacrylamide gel electrophoresis and then transferred onto a PVDF membrane (Bio-Rad, Hercules, CA, USA). The membranes were blocked with 5% non-fat milk overnight at 4°C. Membranes were incubated with primary antibodies at 4°C overnight and then probed with secondary antibody at room temperature for 1 h. The antibodies used in this study are as follows: ARF6 (#ab131261) and PDK1 (#207450) primary antibodies were purchased from Abcam; Glut1 (#12939), HK2 (#2867) and LDHA (#3582) primary antibodies were purchased from Cell Signal Technology; p44/42 MAPK (ERK1/2) (#9102), phospho-p44/42 MAPK (ERK1/2) (Thr202/Tyr204) (#8544) and GAPDH (#MB9231) primary antibodies and horseradish peroxidase (HRP)-labeled goat anti-rabbit or -mouse secondary antibody (#BS13278 or #BS12478) were bought from Bioworld Technology. All other agents were purchased from Sigma (St. Louis, MO, USA).

### CCK-8 and Colony Formation Assays

The proliferative capacity of chordoma cells was determined by CCK-8 assay (Beyotime Institute of Biotechnology; Shanghai, China). Approximately 2 × 10^3^ U-CH1 cells were seeded into each well of a 96-well plate. At the indicated time points, 10 μl CCK-8 solution (Sigma) was added into each well. After 2 h incubation at 37°C, the optical density value of each well at OD450 nm was measured by a microplate reader (Thermo Fisher Scientific) and growth curves were plotted. The experiments were conducted in quadruplicate and repeated three times.

Colony formation was determined by soft agar assay. Briefly, 5000 U-CH1 or U-CH2 cells infected with the indicated lentivirus for 48 h were seeded in medium containing 0.4% soft agar on top of a layer consisting of 0.6% soft agar medium. Cells were cultured at 37°C with 5% CO_2_ for 14 days. Cells were then fixed with 4% formaldehyde and stained with 1 µg/ml ethidium bromide. Colonies (>10 cells) were counted under a microscope in 10 fields per well.

### Dual Luciferase Activity Assay

To examine the regulation between lncRNA MDFIC-7 and miR-525-5p, U-CH1 or U-CH2 cells were co-transfected with pmirGLO-MDFIC-7-WT, which contains the predicted miR-525-5p binding site, or pmirGLO-MDFIC-7-mut, together with miR-525-5p mimic, inhibitor or mock. To investigate the effect of lncRNA MDFIC-7 and miR-525-5p on ARF6, cells were co-transfected with pmirGLO-ARF6-3′UTR-WT and pmirGLO-ARF6-3′UTR-mut, together with miR-525-5p mimic or pcDNA-MDFIC-7. Transfection was performed using Lipofectamine 2000 agent (Invitrogen). After transfection for 48 h, the luciferase activity was calculated using the Dual-Luciferase Assay Kit (Promega) on a GloMax 20/20 Luminometer. Firefly luminescence was normalized to the Renilla luminescence according to the manufacturer’s instructions.

### RNA Immunoprecipitation Assay

To investigate the interaction between lncRNA MDFIC-7 and miR-525-5p, RIP analysis was conducted using the Magna RIP™ RNA-Binding Protein Immunoprecipitation Kit (Millipore, Billerica, MA, USA). Briefly, transfected U-CH1 cells were lysed in RIP lysis buffer. Cell lysates were then incubated with anti-Ago-2 (#SAB4301150, Sigma) or anti-IgG (#I4131, Sigma) in RIP buffer overnight at 4°C. The levels of lncRNA MDFIC-7 and miR-525-5p in the immunoprecipitated RNAs were examined by qRT-PCR.

### Assays on Extracellular Acidification Rate and Oxygen Consumption Rate

To measure the glycolytic capacity and mitochondrial function of chordoma cells, the Seahorse Bioscience XF96 Extracellular Flux Analyzer and Seahorse XF Glycolysis Stress Test (#103020-100, Agilent) and Cell Mito Stress Test kits (#103015-100, Agilent) were used according to the manufacturer’s instruction.

### *In Vivo* Xenograft Tumor Experiments

Nude mice of BALB/c were obtained from the Model Animal Research Center of Nanjing University (Nanjing, China). A total of 10 male mice (4 to 6 weeks old) were randomized in 2 groups (n=5 per group). Approximately 4×10^6^ U-CH1 or U-CH2 cells infected with lentivirus (Lv-sh-MDFIC-7 or Lv-sh-NC) were inoculated into the right flank by subcutaneous injection. Tumors were measured once every 5 days for 35 days using a vernier caliper, and tumor volume was calculated using the formula: length × width^2^ × 0.5. After 5 weeks, the mice were sacrificed and the tumor tissues were collected for weighing and gene expression analysis. The small animal euthanasia equipment was used for laboratory mice euthanasia, and the Euthanasia protocols were followed according to the guideline established by the American Medical Veterinary Association (AMVA) to minimize animal pain and suffering. The animal experiments were approved by the Committee on the Ethics of Animal Experiments of the First Affiliated Hospital of Soochow University.

### Bioinformatics Analysis

The DIANA tool was used to predict the potential interaction between miRNAs with lncRNA MDFIC-7 and the potential target mRNAs of miRNA-525-5p.

### Statistical Analysis

The results are shown as mean ± standard deviation (SD). The data were analyzed using GraphPad Prism 9 (CA, USA) and SPSS software (version 19.0, SPSS Inc., NY, USA). Statistical significance was tested by two-tailed Student’s *t*-test for two group comparisons and one-way analysis of variance (ANOVA) test with post-hoc analysis contrasts for multi-group comparisons. *p*<0.05 was considered significant.

## Results

### LncRNA MDFIC-7 Is Upregulated in Chordoma Tumor Tissues

**Figure 1:**
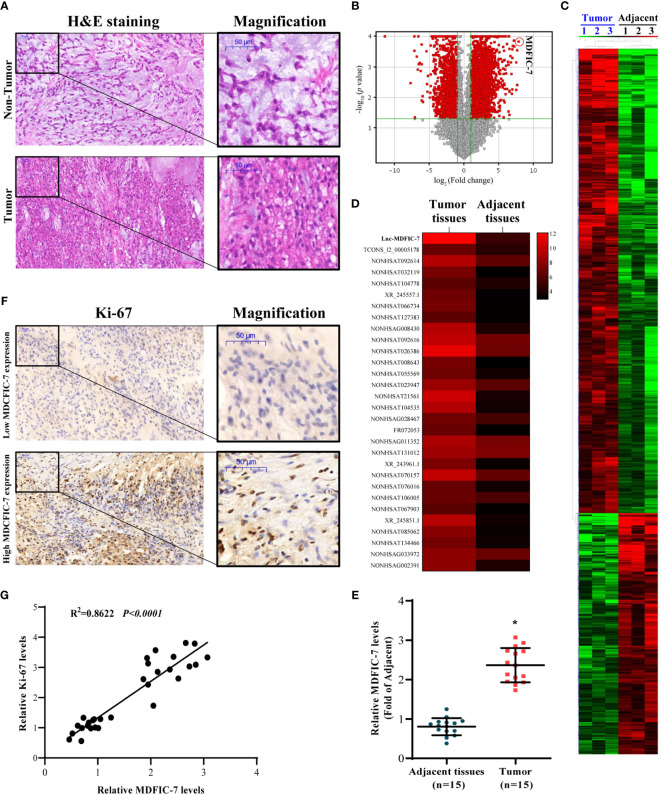
LncRNA MDFIC-7 expression is upregulated in tumor tissues of chordoma. **(A)** H&E staining of clinical chordoma tumor tissues and adjacent non-tumor tissues. **(B)** Volcano plot illustrating fold-change in expression (log_2_ fold change) against statistical significance (-log_10_ adjusted P values) for all lncRNAs in tumor tissues *vs*. adjacent non-tumor tissues from three randomly selected chordoma patients. Red dots represent differentially expressed lncRNAs. **(C)** A total of 4900 differentially expressed lncRNAs are presented in the heatmap (tumor tissues *vs*. adjacent normal tissues). **(D)** Heat map of the expression of the top 30 upregulated lncRNAs in tumor tissues *vs*. adjacent normal tissues (red and black bars represent up- and down-regulation, respectively). **(E)** qRT-PCR analysis of lncRNA MDFIC-7 expression in tumor tissues and adjacent non-tumor tissues. *p < 0.05 *vs*. adjacent tissues. **(F)** Immunohistochemistry of Ki-67 in tumor tissue and adjacent non-tumor tissue from chordoma patients. **(G)** Correlation analysis between lncRNA MDFIC-7 and Ki-67 expression in tumor tissues and adjacent non-tumor tissues of chordoma patients.

We collected tumor tissues and paired adjacent non-tumor tissues from 15 chordoma patients. H&E staining revealed pathological characteristics of the tumor which that distinguished from the adjacent non-tumor tissues (**Figure 1A**). To examine the expression profiles of lncRNAs in tumor tissues compared to adjacent non-tumor tissues, we performed lncRNA sequencing (lncRNA-seq) analysis on three pairs of randomly selected tissue samples. On the basis of the lncRNA fold change, p-values and bioinformatics analysis (**Figure 1B**), a total of 4900 differentially expressed lncRNAs were identified. These lncRNAs were shown in the heatmap in **Figure 1C**. The top 30 upregulated lncRNAs were screened out and shown in **Figure 1D**. Meanwhile, the top 30 downregulated lncRNAs were also screened out and shown in **Figure S1**.

The most significantly upregulated lncRNA was lncRNA MDFIC-7, and we further validated the expression of lncRNAs by qRT-PCR in the chordoma and adjacent normal tissues. The results showed that lncRNA MDFIC-7 was significantly upregulated in tumor tissues compared to adjacent non-tumor tissues (**Figure 1E**). We evaluated the expression of the tumor proliferation biomarker Ki-67 in tumor and non-tumor tissues and identified a significant correlation between lncRNA MDFIC-7 and Ki-67 expression in chordoma tissue (**Figures 1F, G**).

### Knockdown of lncRNA MDFIC-7 Inhibits Proliferation of Chordoma Cells

**Figure 2:**
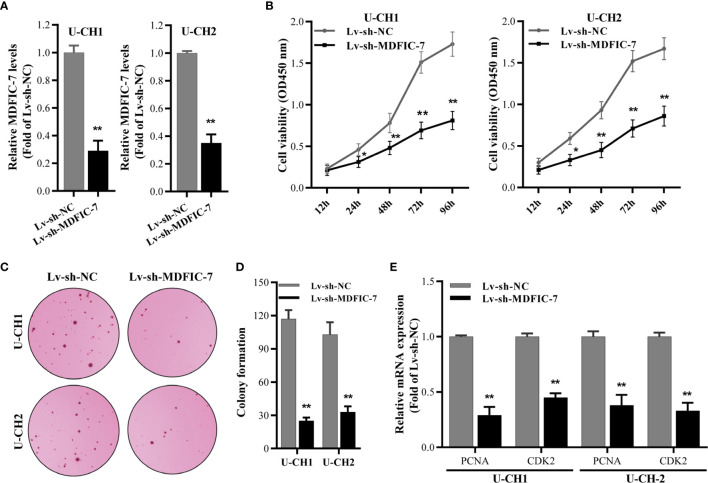
LncRNA MDFIC-7 promotes chordoma cell proliferation. **(A)** qRT-PCR on U-CH1 and U-CH2 cells with lentivirus-induced knockdown of lncRNA MDIF-7 after 48 h. **p < 0.01 *vs*. Lv-sh-NC. **(B)** CCK-8 assay in U-CH1 and U-CH2 cells after lentivirus infection at the indicated time points. *p < 0.05 *vs*. Lv-sh-NC, **p < 0.01 *vs*. Lv-sh-NC. **(C, D)** Colony formation assay in U-CH1 and U-CH2 cells after infection with Lv-sh-NC and Lv-sh-MDFIC-7. **p < 0.01 *vs*. Lv-sh-NC. **(E)** qRT-PCR analysis on mRNA expression of PCNA and CDK2 in U-CH1 and U-CH2 cells after knockdown of lncRNA MDFIC-7 by lentivirus infection. **p < 0.01 *vs*. Lv-sh-NC.

To investigate the role of lncRNA MDFIC-7 in regulating the tumor progression of chordoma, we first inhibited lncRNA MDFIC-7 expression in U-CH1 and U-CH2 cells using lentivirus expressing shRNA-MDFIC7. The efficiency of lncRNA MDFIC-7 inhibition was examined by qRT-PCR and the results confirmed reduced expression of lncRNA MDFIC-7 in cells infected with lentivirus targeting MDFIC-7 (Lv-sh-MDFIC-7) compared with the control (Lv-sh-NC) (**Figure 2A**). Knockdown of lncRNA MDFIC-7 by lentivirus significantly suppressed the proliferation of chordoma cells, as determined by CCK-8 and colony formation assays (**Figures 2B–D**). In addition, knockdown of lncRNA MDFIC-7 resulted in decreased expression of the cell growth biomarkers PCNA and CDK2 (**Figure 2E**). These results demonstrated that lncRNA MDFIC-7 inhibition suppressed the proliferation of chordoma cells and suggested that lncRNA MDFIC-7 functions as a tumor promoter in chordoma by enhancing cell proliferation.

### lncRNA MDFIC-7 Acts as Molecular Sponge of miR-525-5p in Chordoma Cells

**Figure 3:**
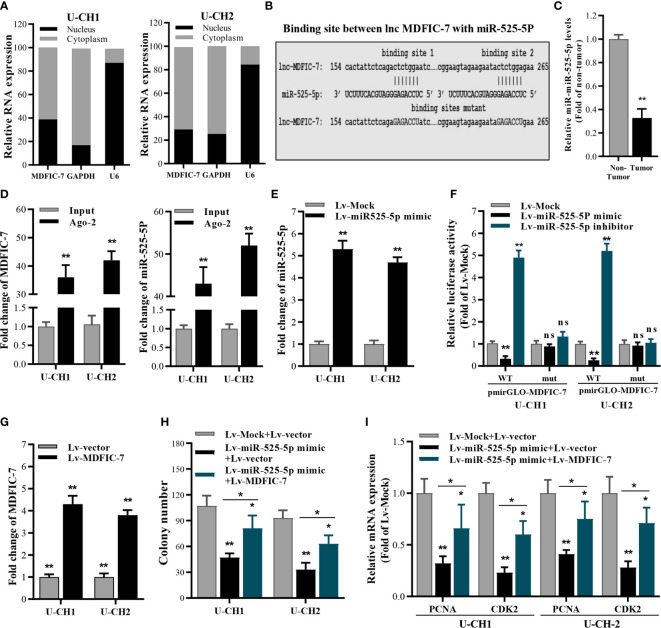
LncRNA MDFIC-7 sponges with miR-525-5p. **(A)** qRT-PCR analysis of lncRNA MDFIC-7 in nuclear and cytoplasmic fractions. GAPDH mRNA and U6 were used as a cytoplasmic control and nuclear control, respectively. **(B)** The predicted binding site between lncRNA MDFIC-7 and miR-525-5p. **(C)** qRT-PCR analysis of miR-525-5p in clinical chordoma tissues. **p < 0.01 *vs*. non-tumor. **(D)** qRT-PCR analysis on the expression of lncRNA MDFIC-7 and miR-525-5p after anti-Ago2-mediated RIP assay in U-CH1 and U-CH2 cells. **p < 0.01 *vs*. input. **(E)** qRT-PCR analysis of miR-525-5p in U-CH1 and U-CH2 cells after infection with Lv-miR525-5p mimic. **p < 0.01 *vs*. Lv-mock. **(F)** Dual luciferase reporter assay in chordoma cells transfected with pmirGLO-MDFIC-7-wt or pmirGLO-MDFIC-7-mut together with mock, miR-525-5p mimic or miR-525-5p inhibitor. **p < 0.01, ns, no significant differences *vs*. Lv-mock. **(G)** qRT-PCR analysis of lncRNA MDFIC-7 in U-CH1 and U-CH2 cells after infection with Lv-MDFIC-7. **p < 0.01 *vs*. Lv-vector. **(H)** Colony formation assay in chordoma cells infected with Lv-miR525-5p or co-infected with Lv-miR525-5p and Lv-MDFIC-7. *p < 0.05 *vs*. Lv-miR-525-5p+Lv-MDFIC-7, **p < 0.01 *vs*. Lv-mock+Lv-vector. **(I)** qRT-PCR of PCNA and CDK2 mRNAs in U-CH1 and U-CH2 cells after infection with the indicated lentivirus for 48 h. *p < 0.05 *vs*. Lv-miR-525-5p+Lv-MDFIC-7, **p < 0.01 *vs*. Lv-mock+Lv-vector.

To analyze the mechanism by which lncRNA MDFIC-7 regulates proliferation of chordoma cells, we examined the subcellular location of lncRNA MDFIC-7 in U-CH1 and U-CH2 cells. The nucleus and cytoplasm of cells were fractionated and the total RNA fractions were isolated and examined by qRT-PCR. The results showed that lncRNA MDFIC-7 is mainly distributed in the cytoplasm of chordoma cells (**Figure 3A**). It was suggested that lncRNA MDFIC-7 could act as a competing endogenous RNA (ceRNA) in human chordoma. The potential target miRNAs of lncRNA MDFIC-7 were predicted by online tools, including DIANA and StarBase 3.0. The results revealed that miR-525-5p contains a putative binding site for lncRNA MDFIC-7, suggesting miR-525-5p as a potential target of MDFIC-7 (**Figure 3B**). We found that the expression of miR-525-5p was decreased in tumor tissues compared with adjacent non-tumor tissues of chordoma patients (**Figure 3C**). We examined the binding between lncRNA MDFIC-7 and miR-525-5p using RIP, and the results demonstrated an interaction between lncRNA MDFIC-7 with miR-525-5p (**Figure 3D**). To further examine the interaction between lncRNA MDFIC-7 with miR-525-5p, we performed the dual luciferase assay. First, the overexpression effect of miR-525-5p mimic by lentivirus infection was detected by RT-qPCR in U-CH1 and U-CH2 cells (**Figure 3E**). The dual luciferase assay results revealed that the luciferase activity of the wild-type MDFIC-7 reporter (pGLO-MDFIC-7-WT) was significantly repressed after the introduction of miR-525-5p mimic, but miR-525-5p mimic had no impact on the mut-lncRNA MDFIC-7 reporter (**Figure 3F**). Furthermore, the activity of the wild-type MDFIC-7 reporter (pGLO-MDFIC-7-WT) was significantly enhanced after the introduction of miR-525-5p inhibitor, but miR-525-5p inhibitor had no impact on the mut-lncRNA MDFIC-7 reporter (**Figure 3F**). These data indicated that lncRNA MDFIC-7 may play a potential role in enhancing tumor progression of human chordoma *via* sponging miR-525-5p.

To further investigate the specific function of miR-525-5p in chordoma cell proliferation, we performed colony formation assay to determine the effect of miR-525-5p on cancer cell growth. MiR-525-5p overexpression inhibited cell growth of U-CH1 and U-CH2 cells, and this effect was blocked by overexpression of lncRNA MDFIC-7 (**Figures 3G, H**). In addition, the mRNA levels of cell proliferation biomarkers PCNA and CDK2 were decreased by miR-525-5p overexpression and this effect was blocked by overexpression of lncRNA MDFIC-7 (**Figure 3I**). These results demonstrated that lncRNA MDFIC-7 acts as a molecular sponge of miR-525-5p and indicated that lncRNA MDFIC-7 and miR-525-5p may play oncogenic and tumor suppressor roles, respectively, in regulating chordoma progression.

### ARF6 Is a Direct Target of miR-525-5p and Is Positively Regulated by lncRNA MDFIC-7

**Figure 4:**
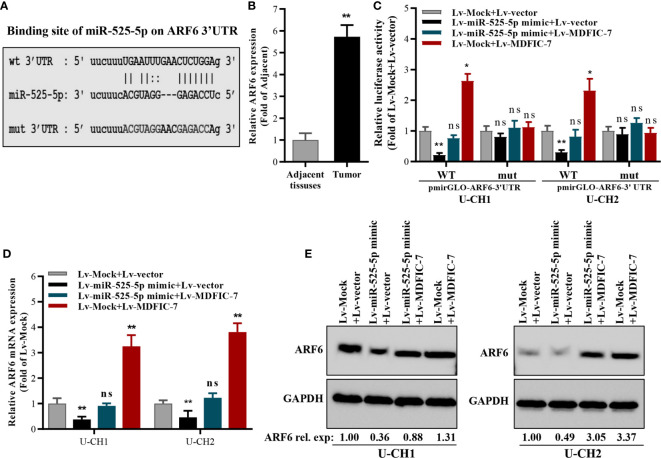
ARF6 is a target of miR-525-5p and is regulated by the interaction of lncRNA MDFIC-7 and miR-525-5p. **(A)** Predicted binding site of miR-525-5p in the ARF mRNA 3′UTR. **(B)** qRT-PCR analysis of ARF6 mRNA expression in chordoma tissues. **p < 0.01 *vs*. adjacent non-tumor tissues. **(C)** Dual luciferase reporter assay in chordoma cells transfected with pmirGLO-ARF6 3′UTR-wild type or pmirGLO-ARF6 3′UTR-mut type reporter plasmids and infected with lentivirus expressing miR-525-5p mock, miR-525-5p mimic or MDFIC-7 as indicated. *p < 0.05 *vs*. Lv-mock+Lv-Vector, **p < 0.01 *vs*. Lv-mock+Lv-Vector, ns, no significant differences *vs*. Lv-mock+Lv-vector. **(D)** qRT-PCR analysis of ARF6 mRNA expression in cancer cells infected with the indicated lentivirus for 48 h. **p < 0.01 *vs*. Lv-mock+Lv-vector, ns, no significant differences *vs*. Lv-mock+Lv-vector. **(E)** Western blot analysis of ARF6 protein expression in cancer cells infected with the indicated lentivirus for 48 h.

We next examined the potential target genes of miR-525-5p by bioinformatics analysis using the online tools DIANA and StarBase3.0. The 3′UTR in the mRNA of ARF6 was predicted to contain a complementary binding site for miR-525-5p (**Figure 4A**), suggesting that ARF6 might be a potential target of miR-525-5p. The expression of ARF6 was evaluated and found increased in chordoma tissues compared to adjacent non-tumor tissues (**Figure 4B**). Dual luciferase reporter assay revealed that miR-525-5p mimic transfection inhibited the activity of the luciferase reporter harboring the wild-type ARF6 3′UTR in chordoma cells, while co-transfection with lncRNA MDFIC-7 blocked the inhibitory effect of miR-525-5p mimic on luciferase activity (**Figure 4C**). However, these effects were abolished with the reporter construct in which the ARF6 3′UTR was mutated (**Figure 4C**). Additionally, RT-qPCR and Western blot assays revealed that miR-525-5p mimic transfection inhibited the expression of ARF6 mRNA and protein in U-CH1 and U-CH2 cells, while co-transfection with lncRNA MDFIC-7 reversed the effect of miR-525-5p overexpression on ARF6 mRNA and protein expression (**Figures 4D, E**). Notably, transfection with lncRNA MDFIC-7 alone enhanced both ARF6 mRNA and protein expression (**Figures 4D, E**). These results showed that ARF6 is a direct target of miR-525-5p, and its expression is positively regulated by lncRNA MDFIC-7.

### ARF6 Overexpression Reverses the Suppressive Effect of lncRNA MDFIC-7 Knockdown or miR-525-5p Overexpression on the Proliferation of Chordoma Cells

**Figure 5:**
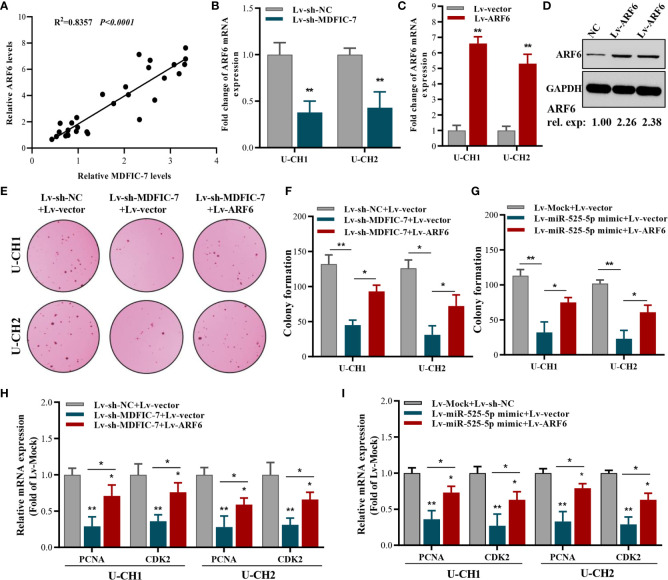
ARF6 overexpression reverses the suppressive effect of lncRNA MDFIC-7 knockdown or miR-525-5p overexpression on cancer cell proliferation. **(A)** Correlation analysis between ARF6 and Ki-67 expression in tumor tissues and adjacent normal tissues of chordoma patients determined by qRT-PCR assay. **(B)** qRT-PCR analysis on ARF6 mRNA expression in cancer cells after lncRNA-MDFIC7 knockdown. **(C, D)** Effect of ARF6 mRNA and protein overexpression in U-CH1 and U-CH2 cells as detected by qRT-PCR and Western blot, respectively. **(E, F)** Colony formation assay in U-CH1 and U-CH2 cells after infection with Lv-sh-MDFIC-7 or co-infection with Lv-sh-MDFIC-7 and Lv-ARF6. *p < 0.05 *vs*. Lv-miR-lncRNA MDFIC-7+Lv-vector, **p < 0.01 *vs*. Lv-mock+Lv-vector. **(G)** Colony formation assay in U-CH1 and U-CH2 cells after infection with Lv-miR525-5p or co-infection with Lv-miR525-5p and Lv-ARF6. *p < 0.05 *vs*. Lv-miR-525-5p+Lv-vector, **p < 0.01 *vs*. Lv-mock+Lv-vector. **(H)** qRT-PCR analysis of PCNA and CDK2 mRNAs in U-CH1 and U-CH2 cells infected with Lv-MDFIC-7 or co-infected with Lv-MDFIC-7 and Lv-ARF6, *p < 0.05 *vs*. Lv-sh-MDFIC-7+Lv-vector, **p < 0.01 *vs*. Lv-sh-NC+Lv-vector. **(I)** qRT-PCR analysis of PCNA and CDK2 mRNAs in U-CH1 and U-CH2 cells infected with Lv-miR-525-5P or co-infected with Lv-miR525-5p and Lv-ARF6, *p < 0.05 *vs*. Lv-miR525-5p+Lv-vector, **p < 0.01 *vs*. Lv-mock+Lv-vector.

To further evaluate the correlation between ARF6 and lncRNA MDFIC-7 expression, we examined the expression of ARF6 mRNA and lncRNA MDFIC-7 in tumor and adjacent non-tumor tissues by RT-qPCR. A significant correlation between lncRNA MDFIC-7 and ARF6 expression in chordoma was found (**Figure 5A**). The expression of ARF6 mRNA was dramatically inhibited by lncRNA MDFIC-7 knockdown in U-CH1 and U-CH2 chordoma cells (**Figure 5B**). To validate whether ARF6 is a downstream targeted molecule of the lncRNA MDFIC-7/miR-525-5p axis, ARF6 was overexpressed using lentivirus in U-CH1 and U-CH2 chordoma cells and its expression was confirmed by RT-qPCR and Western blot (**Figures 5C, D**). Furthermore, we evaluated the effect of the lncRNA MDFIC-7/miR-525-5p axis and ARF6 on cell proliferation using colony formation and RT-qPCR assays. Our results showed that inhibition of lncRNA MDFIC-7 expression or transfection of miR-525-5p mimic repressed the colony forming activity of chordoma cells, but this effect was reversed by co-expression of ARF6 (**Figures 5E–G**). Moreover, the inhibition of PCNA and CDK2 expression induced by lncRNA MDFIC-7 downregulation or miR-525-5p mimic was also reversed by co-expression of ARF6 (**Figures 5H, I**). These results indicated that lncRNA MDFIC-7/miR-525-5p and ARF6 play important roles in regulating the proliferation of chordoma cells.

### The lncRNA MDFIC-7/miR-525-5p Axis Regulates Aerobic Glycolysis of Chordoma Cells by Modulating ARF6 Expression

**Figure 6:**
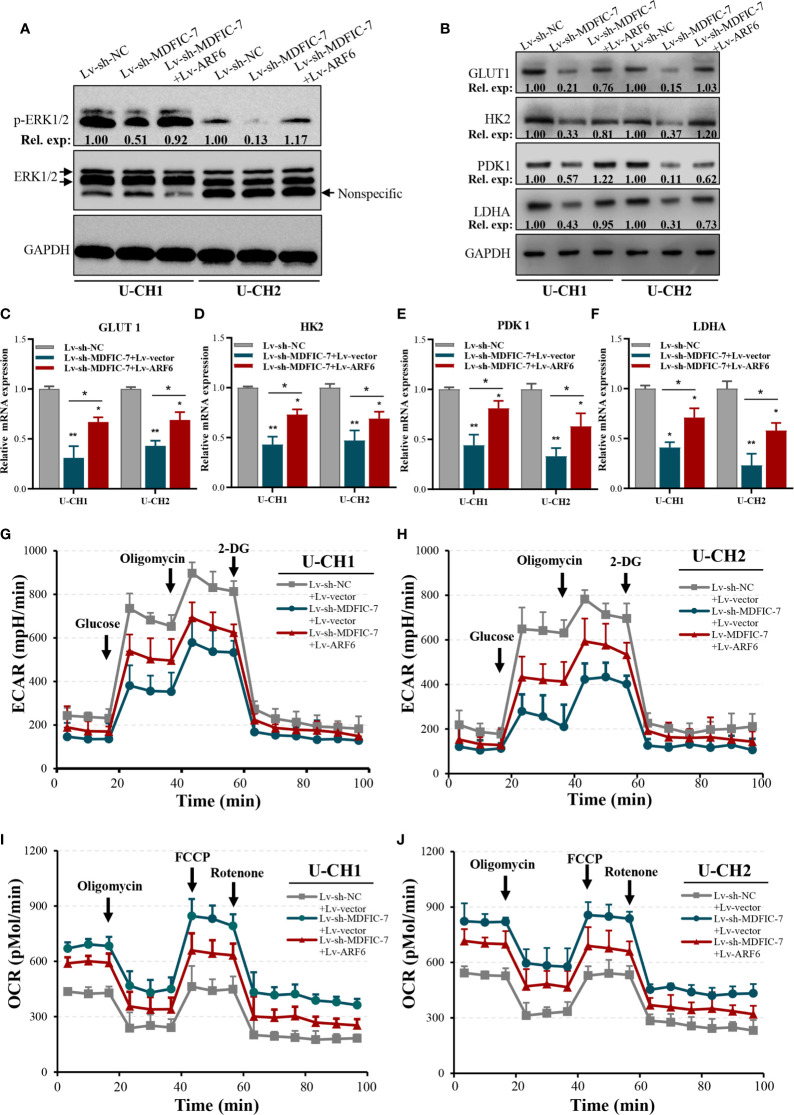
The lncRNA MDFIC-7/miR-525-5p/ARF6 axis regulates aerobic glycolysis of chordoma cells. **(A)** Western blot analysis of phosphorylated ERK1/2 in cells with lncRNA MDFIC-7 knockdown and ARF6 overexpression. **(B)** Western blot analysis on aerobic glycolysis–related proteins, including GLUT1, HK2, PDK1 and LDHA, in U-CH1 and U-CH2 cells with lncRNA MDFIC-7 knockdown and ARF6 overexpression. **(C–F)** qRT-PCR analysis on mRNAs of GLUT1, HK2, PDK1 and LDHA in U-CH1 and U-CH2 cells after infection with Lv-sh-MDFIC-7 or co-infection with Lv-sh-MDFIC-7 and Lv-ARF6, *p < 0.05, **p < 0.01. **(G, H)** LncRNA MDFIC-7 knockdown inhibited the glycolytic capacity of U-CH1 and U-CH2 cells, and ARF6 overexpression reversed the inhibitory effect of LncRNA MDFIC-7 knockdown on cancer cell glycolytic capacity, as reflected by ECAR analysis. **(I, J)** LncRNA MDFIC-7 knockdown increased cancer cell mitochondrial respiration in U-CH1 and U-CH2 cells, and ARF6 overexpression reversed the stimulatory effect of LncRNA MDFIC-7 knockdown on cancer cell mitochondrial respiration, as reflected by OCR detection.

Previous studies showed that ARF6 is a downstream factor in the Kras/ERK signaling pathway and promotes proliferation and the Warburg effect in pancreatic cancer cells (34). To investigate whether ARF6 plays a similar role in chordoma cells and whether this function was modulated by the lncRNA MDFIC-7/miR-525-5p axis, we examined the status of ERK1/2 and the expressions of a series of glycolytic genes that encode key proteins with important roles in metabolizing glucose into lactate that are directly related to aerobic glycolysis, such as glucose transporter type 1 (GLUT1), hexokinase 2 (HK2), pyruvate dehydrogenase kinase 1 (PDK1) and lactate dehydrogenase A (LDHA) in U-CH1 and U-CH2 cells. The results showed that inhibition of lncRNA MDFIC-7 reduced activation of ERK1/2 (**Figure 6A**) as well as the expressions of GLUT1, HK2, PDK1 and LDHA mRNAs and proteins (**Figures 6B–F**). However, these inhibitory effects were reversed by ARF6 overexpression (**Figures 6A–F**). These findings suggest the role of lncRNA MDFIC-7/miR-525-5p/ARF6 axis in ERK signaling activation and aerobic glycolysis.

We further evaluated the changes in aerobic glycolysis in U-CH1 and U-CH2 cells with lncRNA MDFIC-7 knockdown cells using the Seahorse XF analyzers. The ECAR was significantly decreased in lncRNA MDFIC-7 knockdown cells, indicating that inhibiting lncRNA MDFIC-7 suppressed the glycolytic process in chordoma cells (**Figures 6G, H**). Notably, the effect of glycolysis was reversed by overexpression of ARF6 (**Figures 6G, H**). The OCR value reflects glucose mitochondrial oxidation. Consistent with the ECAR results, lncRNA MDFIC-7 knockdown enhanced OCR in chordoma cells, and the promotion of OCR was reversed by ARF6 overexpression (**Figures 6I, J**). These results demonstrated that lncRNA MDFIC-7/miR-525-5p plays an important role in regulating aerobic glycolysis of chordoma cells through modulating ARF6 expression.

### The lncRNA MDFIC-7/miR-525-5p/ARF6 Axis Regulates the Tumorigenicity of Chordoma Cells *In Vivo*

**Figure 7:**
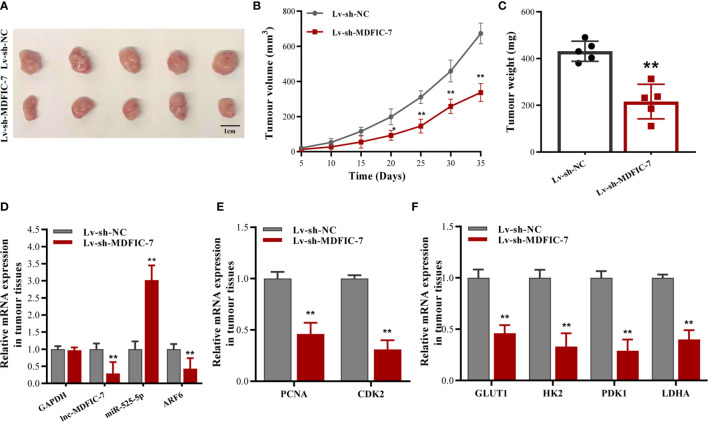
Inhibition of lncRNA MDFIC-7 expression suppresses chordoma cell growth and aerobic glycolysis gene expression *in vivo*. **(A)** Representative images of tumor xenografts collected from lncRNA MDFIC-7 knockdown (Lv-sh-MDFIC-7) and control (Lv-sh-NC) groups. The tumor volumes were calculated every 5 days after cell inoculation. **(B)** Growth curve of tumor xenografts measured every 5 days. **(C)** Weights of tumor xenografts in the Lv-sh-MDFIC-7 and Lv-sh-NC groups. **(D)** qRT-PCR analysis of lncRNA MDFIC-7, miR-525-5p and ARF6 mRNA in the xenograft tumor tissues. **(E)** qRT-PCR analysis of PCNA and CDK2 mRNAs in the xenograft tumor tissues. **(F)** qRT-PCR analysis on mRNAs of GLUT1, HK2, PDK1 and LDHA in the xenograft tumor tissues. **p < 0.01.

To further investigate the effects of the lncRNA MDFIC-7/miR-525-5p/ARF6 axis on tumorigenicity *in vivo*, we generated a xenograft tumor mouse model using U-CH1 cells infected with lentivirus to downregulate lncRNA MDFIC-7. The tumors in the Lv-sh-MDFIC-7 group were noticeably smaller than the control Lv-sh-NC group (**Figure 7A**). The data of tumor volume and weight in various groups were shown in **Figures 7B, C**. We evaluated the gene expression of miR-525-5p, ARF6, PCNA and CDK2 in the xenograft tumor tissues by RT-qPCR (**Figures 7D, E**). We also examined the expression of a series of glycolysis-related genes, including GLUT1, HK2, PDK1 and LDHA genes, in the tumors. Consistent with the *in vitro* data, inhibition of lncRNA MDFIC-7 expression suppressed the mRNA expression of GLUT1, HK2, PDK1 and LDHA in the xenograft tumor tissues (**Figure 7F**). These data validated that the lncRNA MDFIC-7/miR-525-5p/ARF6 axis plays an important role in regulating cell proliferation and the Warburg effect in chordoma *in vivo* and modulates chordoma progression.

## Discussion

Chordoma is a type of rare malignant tumor with an incidence of 0.08 per 100,000 individuals (35). The precise mechanism underlying the regulation of chordoma progression remains unknown. Increasing evidence has demonstrated that lncRNAs function as important regulatory molecules in tumor progression, including proliferation, invasion, migration and metastasis, and show dysregulated expression in chordoma tissues (7, 12–15). Here, we identified a new chordoma regulator, lncRNA MDFIC-7, which was upregulated in tumor tissues of patients with chordoma. We investigated the role of lncRNA MDFIC-7 in regulating chordoma progression through various *in vitro* and *in vivo* experiments.

We first clarified the function of lncRNA MDFIC-7 in chordoma cells. Our results demonstrated that lncRNA MDFIC-7 knockdown significantly inhibited chordoma cell proliferation *in vitro*, suggesting that the lncRNA MDFIC-7 exerts tumor promoting activity in chordoma and indicating that lncRNA MDFIC-7 may be a potential prognostic indicator for chordoma.

LncRNAs that are located in the cytoplasm have been shown to function as ceRNAs to sponge miRNAs (36). In our study, we found that lncRNA MDFIC-7 was mainly located in the cytoplasm of chordoma cells, and we identified miR-525-5p as a target of lncRNA MDFIC-7 by bioinformatics analyses and dual luciferase reporter assay. MiRNAs are non-coding RNAs that contain 20–30 nucleotides. MiR-525-5p, a member of the miRNA families, was recently reported to play roles in regulating cell proliferation, invasion, migration, epithelial-mesenchymal transition (EMT) and metastasis in several tumors, including glioma, cervical cancer, ovarian cancer, prostate cancer and colorectal cancer (18, 19, 37–41). These findings indicate that miR525-5p may play an important role in tumor progression. However, no studies have examined the interaction of lncRNA MDFIC-7 and miR-525-5p in chordoma progression. Our findings showed that the significant up-regulation of lncRNA MDFIC-7 in chordoma patient tumor tissues compared with adjacent normal tissues was accompanied by a concomitant decrease in miR-525-5p. MiR-525-5p overexpression inhibited the proliferation of chordoma cells, and miR-525-5p inhibitor reversed the suppressive effect of lncRNA MDFIC-7 knockdown on cancer cell proliferation. These findings demonstrated that the lncRNA MDFIC-7/miR-525-5p axis plays an important role in controlling tumorigenesis and that lncRNA MDFIC-7 exerts an oncogenic activity in chordoma by sponging miR-525-5p.

Previous studies showed that lncRNAs can function as a ceRNA to sponge miRNAs and thus repress downstream target genes that are associated with the regulation of tumor progression (42). Our mechanistic studies identified ARF6 mRNA as a direct target of miR-525-5p in chordoma cells. ARF6 belongs to the small GTPase ARF family and has well-documented roles in promoting cancer cell invasion, migration and proliferation in various types of tumors (25, 27–34). Previous studies suggested that ARF6, as a master driver of tumorigenesis and tumor progression, activates several important signaling pathways (25). Liang et al. reported that silencing the Arf6 gene interrupted the Kras/ERK signaling pathway, thus repressing cell proliferation and the Warburg effect in pancreatic carcinoma cells (34). Our results were in consistent with these findings. The Kras/ERK pathway contributes to endocytosis and recycling of some membrane receptors. Increasing evidence has shown that the Kras/ERK pathway–mediated metabolism reprogramming is a requirement for uncontrolled cell proliferation and maintenance of malignant property, as cancer cells require both sufficient ATP supply and biosynthetic precursors as cellular building blocks (43). These findings suggest that ARF6 has an important role in ensuring a continuous supply of energy and nutrition for cancer cells. In this study, we demonstrated that lncRNA MDFIC-7 knockdown suppressed cell proliferation, tumorigenicity and the Warburg effect (aerobic glycolysis) of chordoma cells both *in vitro* and *in vivo* through inhibiting the expression of ARF6. Our results indicated that the effect of lncRNA knockdown on suppressing tumor progression and aerobic glycolysis of chordoma was achieved by modulating ARF6 expression *via* the lncRNA MDFIC-7/miR-525-5p axis.

This study has several limitations. First, we did not analyze the association between lncRNA MDFIC-7 or miR-525-5p with prognosis of chordoma patients because of the small sample size and inadequate follow-up time. This limitation could be resolved by expanding the sample size and extending the follow-up time in a future study. Moreover, whether other regulatory factors participated in the regulation of the Warburg effect in chordoma cells need further investigation.

In summary, our study provides the first identification of the lncRNA MDFIC-7/miR-525-5p/ARF6 regulatory network in cell proliferation and glucose metabolism in chordoma. Our findings indicate that this axis could represent novel targets for chordoma therapies.

## Data Availability Statement

The original contributions presented in the study are included in the article/**Supplementary Material**. Further inquiries can be directed to the corresponding authors.

## Ethics Statement

The studies involving human participants were reviewed and approved by the ethics committee of the First Affiliated Hospital of Soochow University. The patients/participants provided their written informed consent to participate in this study. The animal study was reviewed and approved by the ethics committee of the First Affiliated Hospital of Soochow University.

## Author Contributions

KC conceived and designed the study. KZ, ZL and ZW performed experiments and KZ drafted this manuscript. ZZ, XS, and XH collected the patients’ samples and performed the experiments. HM and HY collected, analyzed and interpreted the data. KR and KC edited the manuscript. All authors contributed to the article and approved the submitted version.

## Funding

This work was supported by the National Nature Science Foundation of China (grant no. 81802682, 81972104 and 31800154), the Natural Science Foundation of Jiangsu Province (grant no. BK 20180199), the Suzhou Science and Technology Program for People’s Livelihood (grant no. SS201858) and the Natural Science Foundation of Collaborative Innovation Center of Sichuan for Elderly Care and Health (grant no. YLZBZ2002).

## Conflict of Interest

The authors declare that the research was conducted in the absence of any commercial or financial relationships that could be construed as a potential conflict of interest.

## Publisher’s Note

All claims expressed in this article are solely those of the authors and do not necessarily represent those of their affiliated organizations, or those of the publisher, the editors and the reviewers. Any product that may be evaluated in this article, or claim that may be made by its manufacturer, is not guaranteed or endorsed by the publisher.
