# Supplementary material for: Long Non-Coding RNA MDFIC-7 Promotes Chordoma Progression Through Modulating the miR-525-5p/ARF6 Axis
Source: Front Oncol. 2021 Sep 21;11:743718. doi: 10.3389/fonc.2021.743718 (PMC8491581; doi:10.3389/fonc.2021.743718)

Figure 4E

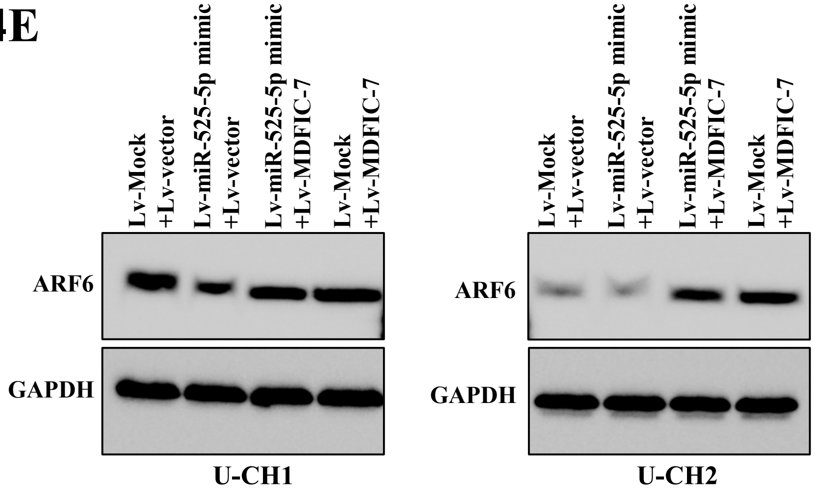

Uncropped WB blot

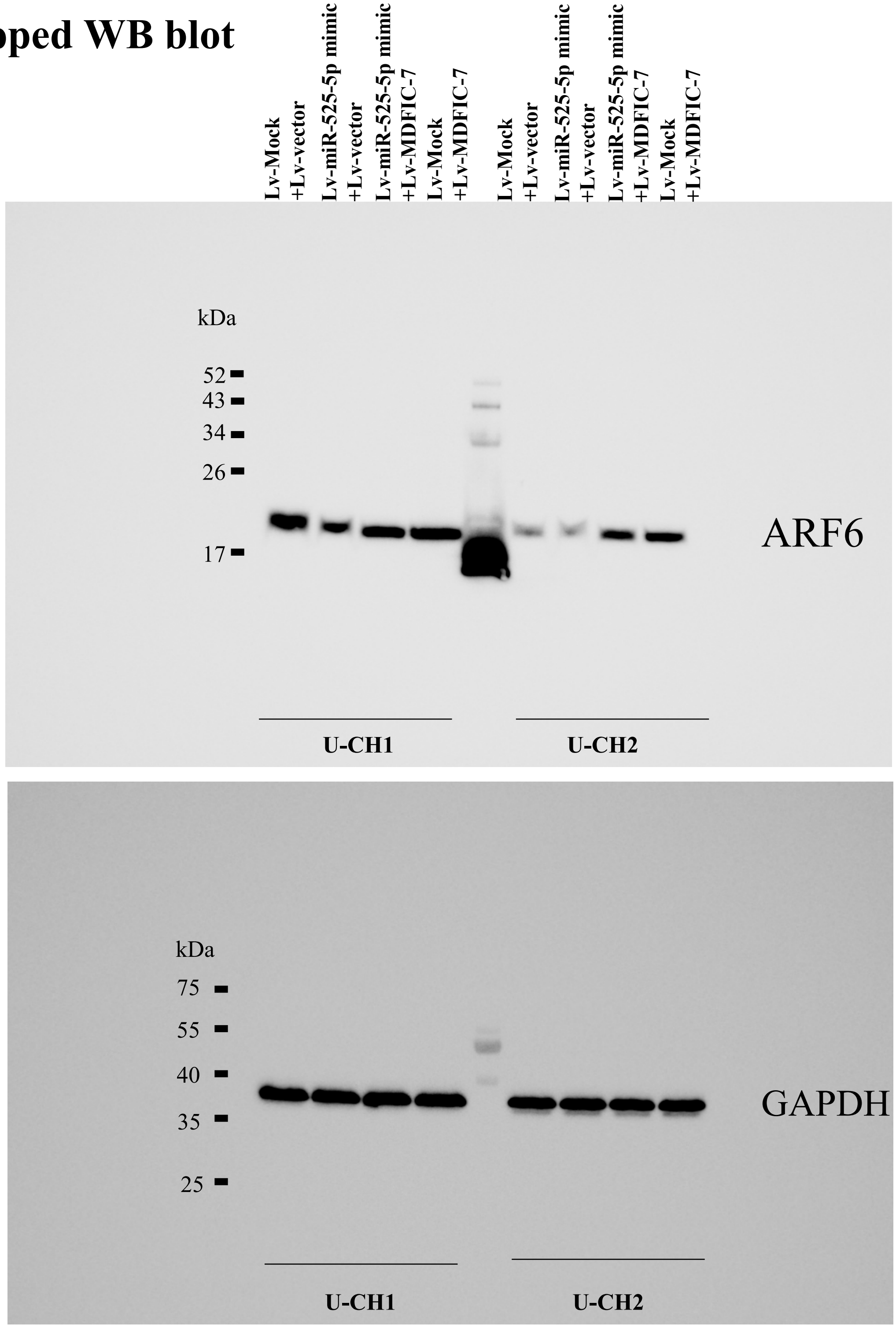

Figure 5D

Uncropped WB blot

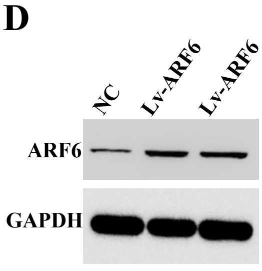

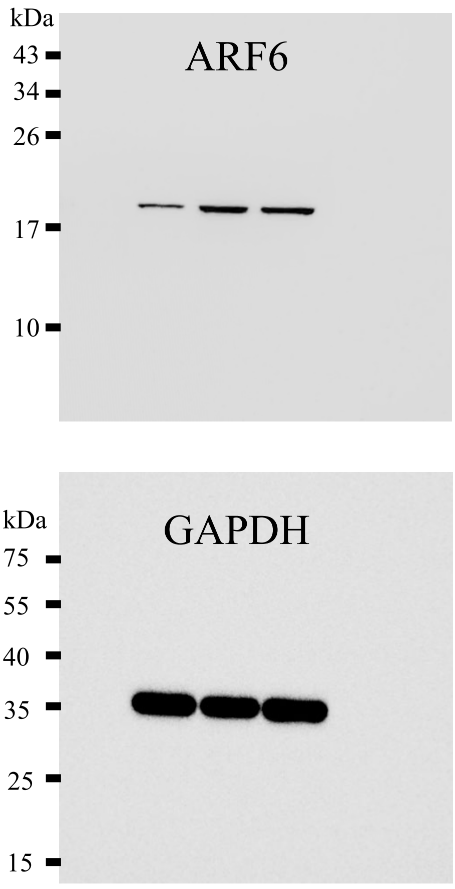

Figure 6A

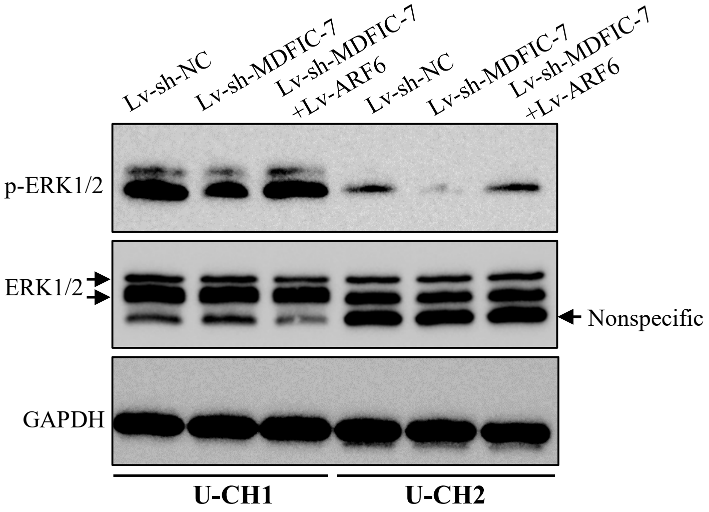

Full uncropped WB Blots

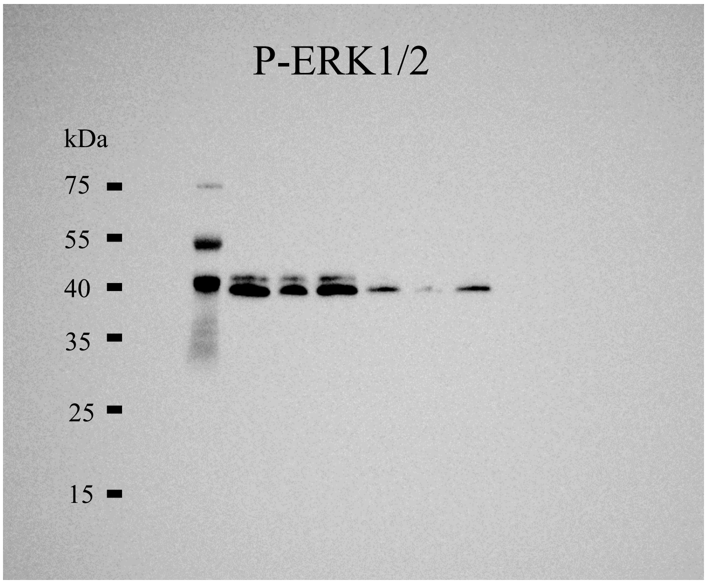

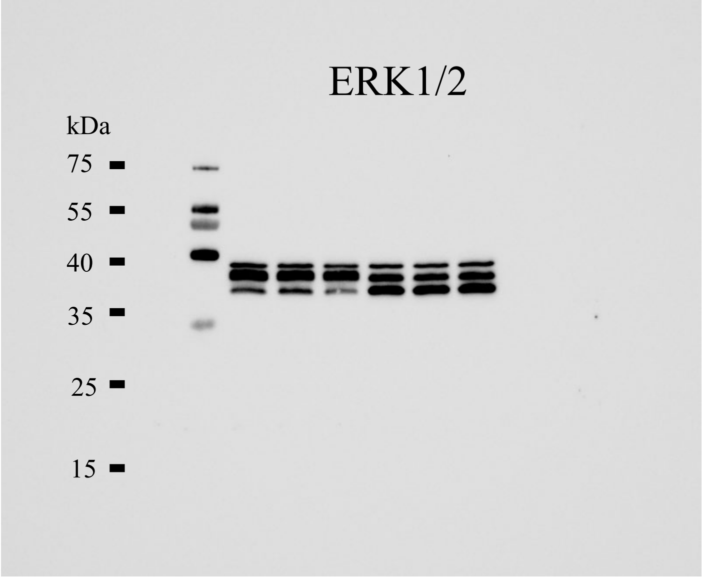

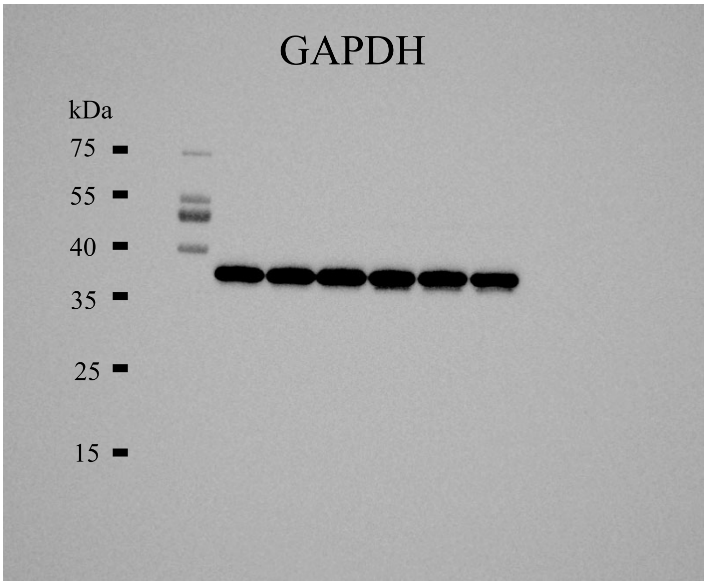

Figure 6B

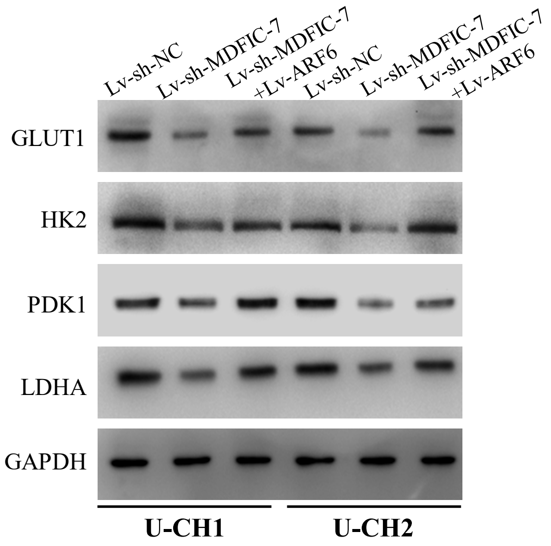

Full uncropped WB Blots

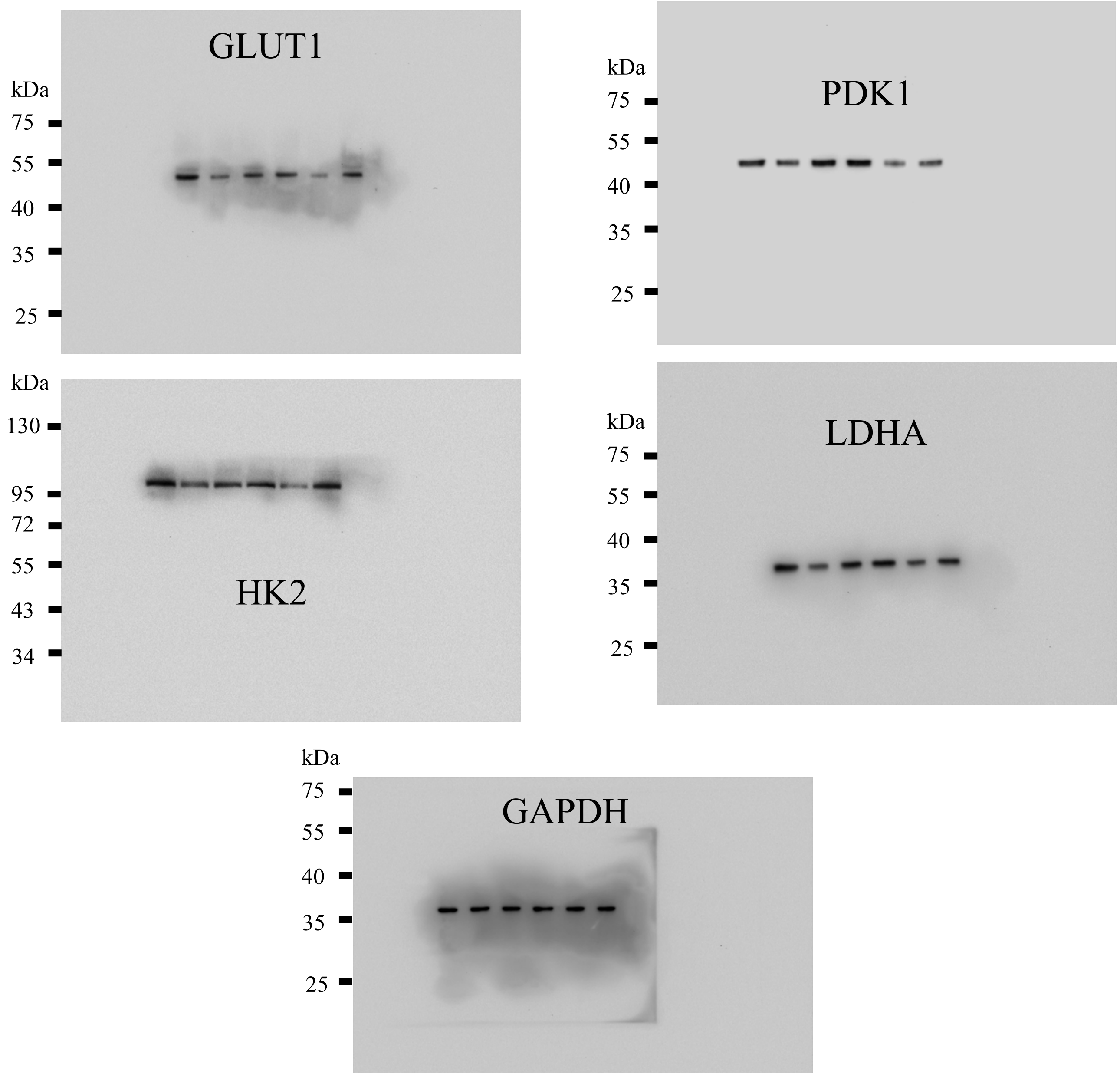

Supplement: Supplementary file 1 [file DataSheet_1.pdf]
