# Supplementary material for: Long Non-Coding RNA MDFIC-7 Promotes Chordoma Progression Through Modulating the miR-525-5p/ARF6 Axis
Source: Front Oncol. 2021 Sep 21;11:743718. doi: 10.3389/fonc.2021.743718 (PMC8491581; doi:10.3389/fonc.2021.743718)
Supplement: Supplementary file 2 [file DataSheet_2.pdf]

# Supplementary materials

## Table S1. Primers for qRT- PCR

| Gene | Forward （5’-3’） | Reverse （5’-3’） |
|---|---|---|
| LncRNA-MDFIC-7 | CCTCACTTGCTGTGGGTGTT | ACTTCCAGGATAGGTCAGGACA |
| PCNA | AACCGGTTACTGAGGGCGAG | AAAGTCTAGCTGGTTTCGGCT |
| CDK2 | CTGCATCTTTGCTGAGATGGTGAC | GAAACTTGGCTTGTAATCAGGCA |
| miR-525-5p | GTCGTATCCAGTGCGTGTCGTG | GCGAGCACAGAATTAATACGACTCAC |
| GLUT1 | TTGCAGGCTTCTCCAACTGGAC | CAGAACCAGGAGCACAGTGAAG |
| HK2 | GAGTTTGACCTGGATGTGGTTGC | CCTCCATGTAGCAGGCATTGCT |
| PDK1 | CATGTCACGCTGGGTAATGAGG | CTCAACACGAGGTCTTGGTGCA |
| LDHA | GGATCTCCAACATGGCAGCCTT | AGACGGCTTTCTCCCTCTTGCT |
| ARF6 | CCAAGGTCTCATCTTCGTAGTGG | AGGTCCTGCTTGTTGGCGAAGA |
| GAPDH | GAAGGCTGGGGCTCATTTG | AGGGGCCATCCACAGTCTTC |
| U6 | CTCGCTTCGGCAGCACA | AACGCTTCACGAATTTGCGT |
