# Supplementary material for: Long Non-Coding RNA MDFIC-7 Promotes Chordoma Progression Through Modulating the miR-525-5p/ARF6 Axis
Source: Front Oncol. 2021 Sep 21;11:743718. doi: 10.3389/fonc.2021.743718 (PMC8491581; doi:10.3389/fonc.2021.743718)
Supplement: Supplementary file 3 [file DataSheet_3.pdf]

Figure S1

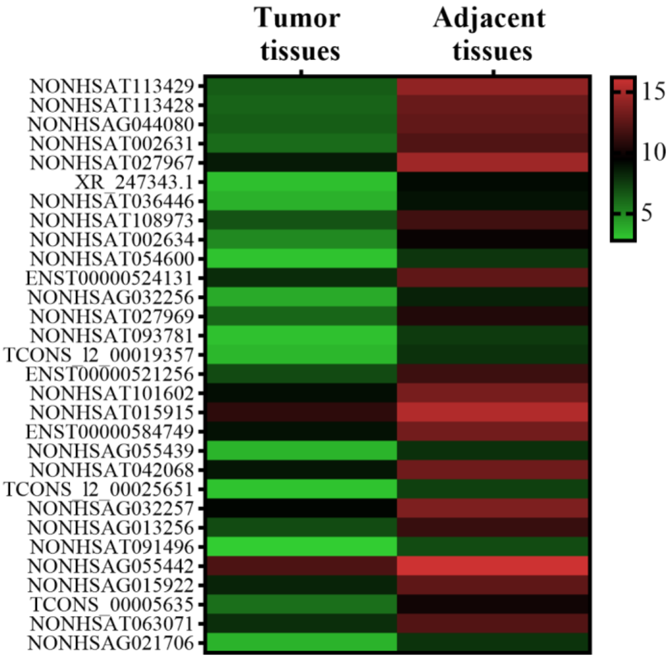

Figure S1 Heat map of the expression of the top 30 downregulated lncRNAs in tumor tissues vs. adjacent normal tissues (red and green bars represent up- and down-regulation, respectively. black bars represent the baseline).
